# Maternal self-conception and mental wellbeing during the first wave of the COVID-19 pandemic. A qualitative interview study through the lens of “intensive mothering” and “ideal worker” ideology

**DOI:** 10.3389/fgwh.2022.878723

**Published:** 2022-09-05

**Authors:** Stephanie Batram-Zantvoort, Lisa Wandschneider, Vera Niehues, Oliver Razum, Céline Miani

**Affiliations:** Department of Epidemiology and International Public Health, School of Public Health, Bielefeld University, Bielefeld, Germany

**Keywords:** COVID-19 pandemic, women's health, self-conception, maternal guilt, public mental health, maternal wellbeing, gender norms, mothering

## Abstract

Mothers tended to be responsible for most of the (additional) caregiving and domestic tasks during the COVID-19 pandemic while simultaneously having to pursue their work duties. Increased role conflicts, parenting stress, and exhaustion predict adverse mental health. We aimed to examine how women referred to and made sense of dominant gender norms in their arrangements of pandemic daily life and how these beliefs impacted their maternal self-conception. Qualitative interviews with 17 women were analyzed through the lens of “intensive mothering” ideology and “ideal workers” norms, emphasizing notions of maternal guilt rising from a perceived mismatch between the ideal and actual maternal self-conception. We found that mothers' notions of guilt and their decreases in health link to dominant discourses on motherhood and intersect with “ideal worker” norms. As such, these norms amplify the burden of gendered health inequalities.

## Introduction

The non-pharmaceutical interventions (NPIs) to contain the spread of the COVID-19-pandemic have unprecedentedly impacted the lives of families with (young) children. Closings of schools and childcare facilities, restrictions on leisure activities, and requirements to work from home called for the re-organization of household tasks, work, and childcare. Mothers tended to be responsible for most caregiving and domestic tasks before the pandemic, and their burden increased during the pandemic (1–4). Evidence shows that parenting stress and exhaustion also increased, especially for mothers of young children (5). Consistent with pre-pandemic data, parenting stress and exhaustion were significant predictors of adverse mental health (6–8).

Adjusting to remote work from one day to the other while in parallel having (young) children to care for at home abruptly aggravated the anyhow “fragile façade of separation that allowed women to be mothers at home and transform into professionals at work” (9). This simultaneity of roles acts as a stressor that has shown to be linked to feelings of guilt when self-evaluating one's performance as a mother and perceiving a discrepancy toward the societally dominant motherhood ideals (10, 11). Feeling ashamed about not meeting self-imposed and societal expectations of being a “good mother” seems to be a universal trait of motherhood that affects stay-at-home mothers and working mothers equally (12). The societal discourse has constructed dichotomous narratives of “good” and “bad” mothering and is accompanied by idealized criteria and unrealistically high expectations toward “good mothering,” also known as *mommy mystique* (13) or *motherhood myths* (14). It is the unattainability of this standard itself that can lead to maternal feelings of guilt (12, 14) and has been linked to adverse (mental) health outcomes, including depressive symptoms and anxiety disorders (15–17).

In the Global North, the dominant ideology of mothering “that all women are disciplined into and judged against” has first been described as *intensive mothering ideal* by sociologist Sharon Hays (10) and recurred to and evolved by various feminist scholars (17–21). *Intensive mothering* has been conceptualized as a gendered model of expectations directed toward mothers by outlining the socially most appropriate way to raise children. As such, this ideology reproduces and manifests gendered hierarchies, stereotypes, and norms (19). Intensive mothering is linked to beliefs that all relate to how mothering is conceptualized, perceived, and lived, among them *essentialism, fulfillment, child-centeredness, challenge, (intellectual) stimulation, and the idea that being simultaneously a caring and working mother is incompatible*.

*Essentialism* assumes that mothers are the most central, critical, and responsible caregivers for the child's development and wellbeing. This, in reverse, justifies blaming mothers for their children's adverse behaviors or developments. *Fulfillment* implies that mothers are at all times satisfied and pleased by their children and their role as a parent, and are not experiencing negative emotions or doubts. Intensive mothering expects mothers to foster their children's cognitive, physical, and social development and organize an environment that is conducive to learning, known as *intellectual stimulation*. The child's (presumed) needs and wishes are superior to the mother's, leading to *child-centered* routines and interactions. This approach is pictured as *challenging* in the sense that it is natural and plausible for mothers to feel exhaustion, yet not leading to question the intensive mothering norms. Last, the intensive mothering norms ideologically *separate mothers from continuing or taking paid professional work*. The underlying belief is that children are so special, pure, and innocent that they deserve to spend their time in the private family sphere in the presence of their mothers (10, 17). These ideals underpin the idea that ‘good mothering' is separated from professional paid work (12, 20, 21).

The *intensive mothering ideology* interacts with and is reinforced by another influential and dominant set of norms described by scholars from the field of work sociology: the *ideal workers' ideology, as a substantial part of gendered organizations* (22–24). Historically, the ideal workers' norms have emerged from the (gendered) separation of the domestic vs. work sphere (25) as a modern phenomenon of economic and societal development after World War II (26). Until today–and despite the significantly increased female labor market participation–subliminal assumptions shape employers and workers expectations and beliefs related to workplace and family roles, favoring masculine ideal worker norms (23).

The ideal workers' norms are constituted of three gendered assumptions: the first assumption builds upon the previously mentioned intensive mothering ideals, especially the idea that children deserve mothers who sacrifice their lives, including their careers, for their children's goods. In other words: mothers prioritize their children over their work obligations (27). The second idea creates an image of the ideal worker who prioritizes work duties over family responsibilities, acts rationally, is fully committed to work obligations, and is strong in leadership (23). Last, the “ideal worker” equates with male employees as female workers (specifically mothers) are perceived as unable to work full-time, believed to be less committed to work, and considered more emotional than rational (23, 27).

Recent findings suggest that the *ideal worker ideology* is applied to and applied by working mothers in terms of career expectations and unwritten penalties, e.g., when women return part-time to work after their parental leave (28). This understanding of “working motherhood” intertwines with the expectations of simultaneously being involved in paid work and fulfilling all caregiving responsibilities, neither of which may be at the expense of the other (29). Conforming with the normative ideals of complementarily being the “good mother” and the “emancipated female worker” is conflicting for many, even though they may have a feminist or gender-equal self-conception (9).

More recent research suggests that women frame mothering and their “working identity” in heterogeneous ways (e.g., by delegating “intensive mothering” tasks or indicating that working leads to being “better” mothers), yet, constantly referring to ideals of “intensive mothering” (21). As such, (working) mothers repeatedly violate the “ideal mother” ideology or the “ideal worker” norms (28).

At the same time, the ideologies of “ideal worker” and “ideal parents” find their counterparts in welfare states' policy measures that support a stereotypical male worker model employed in full-time work over his adult life span on the one hand, and the promotion of a part-time work model for the female primary caretaker on the other hand. The still existing gender pay gap and the gendered distribution of high and low-paid occupations foster parents' negotiations about who is the primary caretaker (e.g., parental leave) and who reduces their own paid work (e.g., part-time work) (28). In Germany and other European countries, norms on masculinity and femininity are highly connected to both working and parenting ideologies and, as such, influence the decision-making processes in the phase of family formation (24): while (expectant) fathers fear disadvantages in their future careers and being perceived as non-masculine or weak, mothers (to-be) fear to be condemned as selfish (30).

During the upswing of the first COVID-19 wave, the closings of schools and childcare facilities abruptly placed families into a context of highly diverging demands (of work, childcare, schooling, and household) that had to be met contemporaneously and concurrently in time and space (5). Women, compared to men, were unequally affected by the additional loads (31) and have shown to be at higher risk of adverse mental health (32) and experiences of overburden (33). While quantitative studies provide evidence that at least some (working) mothers suffer significantly from the pandemic (34), we aim to understand better whether and, if so, how gendered norms and ideologies come into fruition as amplifiers of maternal feelings of guilt or stress. For this purpose, we apply the outlined theories as a lens that guides our further analysis, aiming to fathom how mothers negotiate the “intensive mothering ideology” and “ideal worker” norms in their sense-making. In this regard, we examine the data material through the lens of *intensive mothering* to better understand how the societally dominant mothering approach comes into play in the specific situation of a worldwide pandemic causing substantial changes in families' lives. As the *ideal workers'* ideology dominates work-related norms and identities in the Global North, we are additionally interested in how the women refer to and make sense of these norms in their interviews. Coming from a public health background, we aim to understand better maternal vulnerabilities in terms of emotional disbalance, feelings of guilt or shame, and mental health in relation to their living experiences as mothers and workers.

## Materials and methods

The “Family study” is a COVID-19-specific follow-up of the BaBi birth cohort study established in 2013 in Bielefeld, North-Rhine Westphalia, Germany (35). The Babi cohort study initially explored health disparities in almost 1,000 newborns and their mothers from birth to early childhood. In our Family study, we were interested in the experiences and health of mothers of young children during the time of the first COVID-19 wave in 2020 and the associated NPIs to contain the spread of the virus. We contacted all participants from the BaBi cohort who had previously agreed to be approached again *via* email (*n* = 550) in mid-April 2020 and about 6 weeks later through a reminder to increase participation. The participants were invited to take part in a quantitative online survey (*n* = 124), qualitative email interviews (*n* = 17), or both (*n* = 17). The study has been approved by the Ethics Committee of Bielefeld University (Ref. 2020-059).

### Data collection

We refer in this present article to the qualitative data conducted *via* semi-structured, in-depth email interviews (36). Considering the strict physical distancing measures, closure of childcare facilities, and associated time pressure on the participants, we believed that email interviews would increase flexibility and give the participants more autonomy when they do the interviews (37–39).

The interview process included three waves of open questions that were identical for all participants and, from the second wave on, follow-up participant-specific questions to the answers already provided, aiming to initiate a conversation and deepen the responses. Therefore, in each round, all participants received a set of shared questions and, from round two, additional in-depth, individualized queries. Questions in the first email covered adjusting to the pandemic situation in terms of re-organizing daily life (work, childcare, household obligations) and feelings and experienced ambivalences connected to the participants' role as mothers.

The second wave included questions on the family members' health and wellbeing as well as the share of responsibility in seeking pandemic-relevant information, implementing personal protective measures, and child-orientated communication about the pandemic situation. The third wave finalized this process by asking about views on the future.

### Data analysis

In previous publications on Family study findings, we explored the data through classic content analysis (40, 41). During this process of getting a sense of the data, it became apparent that narratives of “intensive mothering” ideology and “ideal worker norms” seemed to play a role in the self-conception of the women in our sample. The otherwise rather hidden normative ideas that shaped work and motherhood ideals suddenly seemed to become more prevalent and visible due to the major changes families had to face. Therefore, we re-examined the data, this time through directed content analysis. Directed content analysis allows to validate or conceptually extend existing theories (42). We aimed to identify implicit or explicit expressions, narratives, or beliefs relating to the “intensive mothering ideology,” “ideal worker norm,” and expressions of maternal guilt in the interview material. As the emergent pandemic situation required massive adjustments from families in terms of work- and care organization, we were interested in if–and how–societal norms appeared in women's self-conceptions concerning their role as mothers and workers. We coded the interviews according to the elements of the “intensive mothering ideology” (*essentialism* and *fulfillment, challenging, child-centeredness, and (intellectual) stimulation*) and the “ideal worker norm” (*women's prioritization of children over work obligations; ideal workers' prioritization of work over family duties and acting rationally; men's equation with ideal worker norms*). We refrained from using the “challenge” element as an independent coding and analysis unit since the notion of challenge is running through all others aspects of the intensive mothering. The interview passages sharing the same code were carefully re-read, compared, and juxtaposed, aiming to identify shared meanings and similar (just as disparate) ways of how motherhood and work ideals channeled and became present in the women's self-conception and approaches to work and childcare. From this circular data analysis and coding process, we derived the themes presented in the results section.

Drawing on theories for directed analysis presents a caveat, namely a tendency to identify evidence supporting the theory rather than deconstructing it (42). Being mindful of this risk, we tried to be reflective, allowing alternative or deconstructive interpretations of the data material. This has led us, for example, to identify a representation of “ideal worker” norms in the unexpected maternal self-conception of the homemaker.

To synthesize our theory-guided data analysis and place it in the context of the pandemic, we further examined our findings, looking for variations in expressions of maternal self-conception (enhancement, continuation, or deterioration) and its potential relations to maternal guilt (or the absence thereof). We reflected on whether the abrupt changes in daily life have impacted maternal self-conception and, if so, how those shifts related to “intensive mothering” ideology or “ideal worker's norms”.

## Results

### Sample description

Seventeen women participated in the email interviews. Our sample is characterized by highly educated, white middle-class, cis-gender women, all living with their male partners. Most women (*n* = 9) had two children under 18 years living in their household, whereas five women had three or more children, and three women had one child. Most children visited childcare facilities before the onset of the pandemic. Only one child was in so-called “emergency childcare” during the early phase of the pandemic (“emergency care” was available only to those children whose parents both worked in “essential” domains, e.g., health care workers, food, energy, and water supply, teachers). Four other families used emergency childcare when the access criteria were extended (Tables 1, 2). Four out of 17 women were currently out of work (homemaker, parental leave), and the remaining women worked part-time. All male partners were in work, with the majority (*n* = 12) working full-time. Overall, most interviewees drew lines of comparison between the time before the pandemic and their present situation. All women experienced significant changes in their daily lives due to the NPIs, with two trends emerging: one group expressed a positively perceived deceleration of life, while another group felt extremely stressed due to the absence of facility-based childcare and the continuation of their work duties. As to be expected, the latter group experienced a deterioration of wellbeing and mental health. In contrast, the women in the first group felt relaxed and were grateful for the extra quality time with their families.

**Table 1:** Participants characteristics.

	***n***	**valid %**	**mean**	**SD**	**missing (*n*)**
Age	17		37.76	4.21	
Marital status					
Single	0	0.00			
Partnered/married	17	100.00			
Children (> 18) in own household					
1	3	17.65			
2	9	52.94			
3	3	17.64			
4	1	5.88			
5	1	5.88			
Facility-based childcare for children (>7) (cumulated, pre-pandemic)					
None	4	12.50			7
Childcare center	23	71.88			
Nursery	2	6.25			
Other facility	2	6.25			
Child in “emergency care” (during early phase of pandemic)*					
Yes	1	5.88			
No	16	94.12			
Hours spend on housework (pre-pandemic)			12.53	8.17	
Partners' hours spend on housework (pre-pandemic)			5.35	3.50	
Hours spend on housework (during pandemic/ past 2 weeks)			14.06	8.61	
Partners' hours spend on housework (during pandemic/ past 2 weeks)			7.56	4.60	1
Care hours/ week for family members (pre-pandemic)			45.65	27.56	
Partners' care hours/ week for family members (pre-pandemic)			29.59	29.26	
Care hours/ week for family members (during pandemic/ past 2 weeks)			68.20	35.45	2
Partners' care hours/ week for family members (during pandemic/ past 2 weeks)			48.53	37.49	2
Time spend on homeschooling (pandemic)			2.75	1.50	13
Partners' time spend on homeschooling (pandemic)			0.33	0.58	14
Employment status					
In work	13	76.47			
Out of work	4	23.53			
Mode of employment					
Full-time					
Part-time	13	76.47			
Parental leave	2	11.76			
Not employed (e.g., home-maker, student)	2	11.76			
Marginally employed/ state benefit	0	0.00			
Employment status partner					
In work	17	100.00			
Out of work	0	0.00			
Mode of employment partner					
Full-time	12	70.59			
Part-time	4	23.53			
Not employed (e.g., home-maker, student)	0	0.00			
Marginally employed/ state benefit	1	5.88			
Essential worker					
Yes	8	53.33			2
No	7	46.67			
Essential worker partner					
Yes	6	35.29			
No	11	64.71			
List of professions of interviewees	Management assistant, assistant, civil servant, appraiser, controller, data analyst, information technology, teacher, psychologist, psychotherapist, language therapist, in education, student, administration				
Changes in employment situation due to pandemic					
Home-office	5	38.46			
Short-work, mandatory leave	3	23.08			
No changes	3	38.46			4

Source: Quantitative data from Family study.

^*^Quantitative data collection took place 2 weeks before the email interviews. In the meantime, more sectors were declared “essential,” explaining the differences in data regarding the number of children qualifying for “emergency childcare”.

**Table 2:** List of interviewees by name, information derived from email-interviews.

**Interview** ^**a**^		**Occupation**	**Children**	**Childcare situation**	**General impression related to overall wellbeing, mental and physical health (derived from overall email interviews)**
1	Hanna	homemaker, partner: 80% full-time job, 20% self-employed	5, pregnant	At home	More relaxed, more satisfied, more cheerful, relieved, occasionally more stressed
2	Mary	part-time job as essential worker, partner: full-time job	1	Emergency childcare	Less time pressure, more balanced
3	Janine^b^	homemaker, partner: full-time job	4	At home	Some uncertainty, somewhat stressed by taking on childcare responsibilities
4	Jane	maternity leave (for expectant mothers), partner: self-employed, part-time, mainly in home-office	2, pregnant	At home	Strongly physically, nervously, and emotionally stressed, overwhelmed
5	Eve	soon starting job, partner: marginally employed/ state benefit	2	At home	More emotionally stable, more patient, emotionally in a positive mood, less daily stress
6	Karen	homemaker, partner: full-time job, currently in home office	3, pregnant	At home	More time for self-care, gratitude, calmer, more balanced
7	Vanessa	self-employed, partner: full-time, currently in home-office	2	At home	Sometimes somewhat unbalanced, experiences herself as insufficient
8	Sofie	part-time job in home-office, partner: shift work, part-time studies	2	At home	Deceleration, positive perception, and appreciation of one's situation
9	Tina	part-time essential worker, partner: self-employed	2	Emergency childcare (partially)	Less stress, deceleration, feeling happy
10	Lea	part-time essential worker, partner: full-time in home-office	2	Emergency childcare (partially)	Some family-to work-conflicts, more relaxed due to fewer appointments, exhausted but still appreciative view of time spent together with children, stronger migraines
11	Beccy^b^	part-time job in home-office, partner: full-time job (short-time working) in home-office	2	At home	Positive perception and appreciation of one's situation, home-office more stressful but fewer daily stressors
12	Dana	part-time job, partner: full-time job	1, pregnant	Emergency childcare	Hopelessness due to financial situation, worried about child's needs, high stress, psychosomatic symptoms
13	Kate	part-time job in home-office, partner: full-time job in home-office	2	At home	Highly stressed due to work and care duties, frustration, stronger migraines
14	Fiona^b^	part-time job, partially from home-office, partner: full-time job in home-office	2	Emergency childcare (partially)	Unbalanced due to daily monotony
15	Helen	home-office, partner: full-time in home-office	2	At home	Predominantly happy, sometimes irritable, grateful for the privileges of the family
16	Julie	part-time job in home-office, partner: full-time job in home-office	3	At home	High stress level, irritability, psychosomatic symptoms
17	Bianca	part-time job in home-office, partner: full-time job in home-office	1	At home	Uncertainty, more worries

^a^ Names do not correspond to the real names of the interviewees.

^b^ Not quoted in this article (as responses were too short, e.g., in a quantitative survey response style).

### Intensive mothering

We find miscellaneous references to the intensive mothering beliefs of essentialism, fulfillment, stimulation, child-centeredness, and the intersections of mothering and working. Both the themes “essentialist maternal identities” (*n* = 8) and “continuous responsiveness” (*n* = 4) were identified for essentialism. Feeling like a “better mother” (*n* = 6) and “fulfillment outside of mothering” (*n* = 1) refer to the narrative of fulfillment, whereas “pedagogical parenting” (*n* = 5) reflects the idea of stimulating the children's cognitive development. Expressions of strong child-centeredness were labeled as “completely aligned with children's needs” (*n* = 2), and the juxtapositions of mothering and working (in-home office) was visible in five interviews. Three of these felt that they could do no justice to either of them, whereas two interviewees did not experience contradictions between their identities as mothers and workers.

#### Essentialism I: Essentialist maternal identities

Eight interviewees see themselves as their children's primary and most essential caregiver. This manifests in statements such as from Jane, who shares the care work with her partner. Yet she remains the responsible parent when it comes to nursing and the emotional needs of their children and “thereby take[s] over significantly more” (Jane, I: 4). Just as for Jane, Vanessa perceives herself as the parent that takes up most of the emotional and relational aspects of parenting:

“Even though my partner is currently at home more often than I am and is, therefore, more often available for the children (…), I more often take over the ‘emotional' tasks (e.g., conflict resolution, motivation for schoolwork) because the children (and my guilty conscience as a mother) ‘demand' this (…)” (Vanessa, I: 7).

Vanessa is aware that their share of (parenting) tasks is unevenly distributed. Yet, she feels ambivalent since, as opposed to her mind, her “feeling” says that the division is justified or that [she] should rather take on even more tasks.” (Vanessa, I: 7). Asked about the drivers of this ambivalence, she critically evaluates her essentialist self-conception:

“(…) my ambivalence has primarily something to do with the demands I put on myself as well as dysfunctional assumptions (‘I have to be a perfect mother', ‘I earn less money than my partner, so I have to do more in household chores and raising children, ‘I can't expect too much from my partner')” (Vanessa, I: 7).

While for Vanessa and Jane, their greater involvement in absorbing their children's emotional needs is perceived as unfair, they admit that they feel a great sense of responsibility that leads to more involvement compared to their partners. Here, essentialism as part of the *intensive mothering* ideology comes into play in determining Vanessa's and Jane's mothering practices while at least partly contradicting their attitude.

In contrast, Karen presents an essentialist idea of motherhood in unity with her maternal self-conception. She strongly values how much her younger children enjoy staying at home because, in her idea, this constantly provides them the feelings of nesting, familiarity, and safety and advances her children's developmental growth (Karen, I: 6).

Hanna, who deliberately chose to be a stay-at-home mother to her five children, shares the love and fulfillment that Karen has articulated. She clearly expresses her satisfaction with the fact that her and her partners' pre-pandemic lifestyle and role-sharing seamlessly fit the pandemic situation as “what [she holds] in values and [has] practiced before is now coming to fruition”:

“We don't have to make an excessive adjustment: being a mother at home with the children, living with them and providing a reasonable daily structure, values, encouragement and relationship skills” (Hanna, I:1).

She derives her approach to mothering as a higher divine order that corresponds to her Christian religious beliefs:

“We are of the opinion and have also made the experience that life itself and especially life in partnership and family ‘works' most healthily, satisfactorily, happily and effectively when it is lived within the framework of a certain ‘order' (Hanna, I: 1).

Hanna's values firmly attach to a religious and essentialist interpretation of ‘intensive mothering' that she understands as being present and sharing life with her children, guiding them through childhood, and imparting her beliefs and norms into their lives.

#### Essentialism II: Continuous responsiveness

As the ideal of being the primary carer is an integral part of a the “intensive motherhood ideology,” the concept of ongoing maternal responsiveness corresponds to this as an interactional counterpart. Due to the juxtaposition of working, self-care, and childcaring during the lockdown, most mothers in our sample experience the expectation of continuous responsiveness as a burden. For Kate, who has now been working and caring for the kids at home for around 9 weeks, chronic migraine attacks have increased to twice a week; she feels exhausted and has developed gastrointestinal problems. She states the relevance of self-care for her health and wellbeing. She connects her current lack of self-care to the assumption that “mothers must always be responsive” (Kate, I: 13). Even if she rests for just a moment, her children cannot comprehend the fact that she is unavailable as they have never learned that she as a mother might be nearby but not approachable:

“And even when I take time, I often get (..) interrupted because ‘mothers always have to be approachable'. Even though we have explained to our 5-year-old that mom wants to be undisturbed from time to time, he can't understand why he shouldn't address me when I am in the house after all.” (Kate, I:13).

Where Kate experiences a deterioration of her physical health, Hanna notices slight decreases in her wellbeing by the fact that she is permanently present to and requested by her children. Just as Kate, she longs for a break from the continuous expectation of maternal responsiveness that reflects her reality during the pandemic. Jane, who is currently pregnant, perceives the situation as “extremely strenuous” and straining the relationship with her children. She feels that she can neither do justice to her children, as she gets quickly irritated and then reacts inappropriately or in an unfair manner, nor to her unborn child, as she does not find time to brace herself for birth and engage with her baby. She would feel relieved having “more me-time not just being a mother.” Asked about whether she feels more balanced than in the past, she replies that “overload, anger, sadness, and a guilty conscience have clearly increased,” making her feel ashamed and greatly dissatisfied as she currently experiences herself as “a mother, which [she] actually do[es] not want to be at all.” (Jane, I:4).

Julie feels challenged by the intersection of work and care duties. She thinks she would be a “better mother with more freedom to take care of the children” with fewer time constraints. Still, she also indicates that she “[misses] the time without the children, whether concentrated at work or actually alone at sports (or just alone shopping, the main thing is alone!).” (Julie, I:16).

We find that essentialism as a crucial element of the intensive mothering ideology is present in our data in how mothers perceive themselves as the most critical and responsible parent for the positive development of their children and in the actions they took to fulfill this ideal.

All women feel challenged by the (social) expectation of constant maternal attention and responsiveness they need to direct toward their children. Kate reflects how societal expectations are present in her own family dynamics while (apparently) feeling the dilemma of fulfilling these expectations at the expense of her own health. Before the pandemic started, she seemed to have established an equitable share of time for “mothering” and time for herself that is now off balance. Still trying to meet the ideal of being present and available impacts her wellbeing negatively and makes her question her mothering qualities. Jane and Julie, in contrast, make more explicit representations of feeling guilty about their current performance as a mother in depicting their actual self as non-ideal. Interestingly–and in contradiction to the ideal mothering norms of prioritizing the children's needs over the mothers' needs–Kate, Jane, Julie, and Hanna clarify (either explicitly or implicitly) that first of all, they have needs that do not equal to them being mothers (e.g., having alone time) and that meeting those needs contribute to their wellbeing and emotional balance. It seems that they perceive a discrepancy between their “ideal” and the “actual” mothering self because of a lack of time in *not performing* their mother role.

#### Fulfillment I: Feeling like a “better mother”

An approximation to the mothering ideal of fulfillment in their parental role was found in other interviewees, for example, with Tina. As all leisure activities have been canceled, Tina feels relieved due to the absence of afternoon appointments and fills this gap with fun activities like biking, hiking, or building a tipi, making her feel like a “better mother”:

“I can perform the role better. I feel like I can do more justice to being a mom. I didn't expect to enjoy all this free time with the kids so much.” (Tina, I: 9).

Similar to Tina, Hanna and Eve see a connection between their emotional wellbeing and the quality of interactions with their children. Eve states that she has become “more patient and therefore more emotionally stable” because spending time and playing with her children does her good and makes her feel “emotionally re-charged” (Eve, I: 5). Hanna reflects that her having more leisure time leads her to view her “children better in their peculiarities and developments” (Hanna, I: 1). Just as Hanna, Karen and Lea feel profound gratitude about the closeness they currently sense with their children. Karen is grateful for the fact that she can spend “so much time with [her] children and see exactly how they develop “(Karen, I: 6). Besides her essentialist view on her providing the best conditions for her children to develop and grow, she is also completely fulfilled with and merged into her role as a mother:

“I love being a mom, and I also love being a mom around the clock all the time, like I am now in this exceptional situation” (Karen, I: 6).

Lea reflects that she can respond better now to some needs: whenever she feels challenged by her children, she reminds herself “that this time is also finite and that [she] can see it as a gift” (Lea, I:10).

#### Fulfillment II: Fulfillment outside of mothering

Dana and her child were in mother-child cure when the NPIs were implemented; subsequently, she spent a few weeks at home. Therefore, she had a long break off work, which often made her “feel that [she] was doing little that was meaningful” as she didn't enjoy spending most of her time exclusively with her son and “had the impression that [she] couldn't meet his needs” (Dana, I: 12). Now, that she continues to work and her son attends the “emergency” childcare, she feels emotionally more balanced because of the “change in daily structure” and the “task” she now has (Dana, I: 12). However, she still struggles with the sentiment of not meeting her sons' needs adequately, and, along with this, is very concerned about how she will manage two children in the future. Having her child in childcare again seems to be a relief to her as she believes that he is in good hands and receives stimuli that positively foster his development (Dana, I: 12). In her interview, Dana refers to a lot of situations where she enjoyed time with her son and family. Still, she does not relate to the ideal of being “fulfilled” through mothering, nor is she essentializing it. Instead, she locates her sense-making into her working identity and expresses how her work obligations positively influence her wellbeing.

#### Intellectual stimulation: Pedagogical parenting

Vanessa worries about whether she can do justice to promoting her children's needs so they do not miss out (Vanessa, I: 7). Hanna expresses her feeling of being responsible for the children's stimulation by saying that now, her children have fewer out-of-home activities, she must increasingly “provide sufficient activity opportunities (...)” (Hanna, I: 1). Sofie, whose older child is in distance learning, but does not receive material from the school, feels liable for preparing teaching material and additionally enhancing her younger daughter's cognitive progress by providing “interesting exercises, puzzles or painting tasks so that she does not feel neglected” (Sofie, I: 8). Kate feels extremely challenged by needing to perform as a teacher to her child as it opens a role conflict between her being a mother and a ‘teacher'. Yet, she considers it her mission to motivate her child, although she lacks “the pedagogical and didactic skills to always act correctly according to the situation” (Kate, I: 13).

Intellectual stimulation in the sense of actively organizing an environment that fosters the child's development presents another element of ‘intensive mothering ideology'. Kate, Sofie, Vanessa, and Hanna share the attitude of being responsible for their children's development by providing the required tools and learning material. While these women at least subliminally present the efforts as an additional task and somewhat a burden, Helen is enthusiastic about doing more educational work, especially “rules” and “rituals” (Helen, I: 15), for which before the pandemic, she only found time marginally or during weekends.

The analysis of the data material shows that these mothers refer to the “intensive mothering” ideal of stimulation in their educational efforts. The pandemic has re-turned this responsibility into the mothers' sphere of action as school, kindergartens, and afternoon activities have been canceled. In filling this gap, only Helen seems to rise and come closer to her “ideal” of mothering.

#### Child-centeredness: Completely aligned with the children's need

The motherhood ideal of child-centeredness reflects itself into planning routines, activities, and daily tasks around the (presumed) needs of the child, while the parent's needs fade to the background. Eve reveals in her interview a strongly child-centered approach to parenting, whereby she consistently speaks of herself and her husband (‘we'). She describes how the closure of childcare facilities has led to the fact that they can fully accommodate their children's wishes and needs now:

“We were able to respond directly to our little daughter's wish–to become diaper-free. There was no time pressure at all. It did her an incredible amount of good. Also, it was last week when she was ready to give up the pacifier. We have time to be there for her, to accompany her.” (Eve, I: 5).

Eve's use of terminology refers to her parenting approach of planning around the needs and wishes of her children. This child-centeredness is evident in her description of the pandemic-related changes as well:

“Everyday life was completely decelerated. No kids' gymnastics for the big one, no kids' gymnastics for the little one, no music classes, and no more appointments. In general, you simply have time for your children. Nothing is more important” (Eve, I: 5).

Although she says that there now is nothing more important than the time they spend with their children, her descriptions of the pre-pandemic daily life were just as child-centered since she mentioned activities exclusively for her children. The organization of everyday life around children's needs and desires continues in Eve's presentation of their day structure: all household duties, including shopping, cleaning, and cooking, happen during the children's sleeping time, whereas during the daytime, she and her partner fully concentrate on their children's wishes. A comparable child-centered parenting approach was found in Karen, whose children freely decide which parent takes up care duties for them unless her partner is in a video call and therefore, she is the only one available (Karen, I: 6).

“Our two youngest (4 and 2 years old) are still diapered and want their dad to take over when he is home. Our children decide for themselves who they need something from (…)”. (Karen, I: 6).

#### Juxtaposition of mothering and working I: Not doing justice to both mothering and working

An inherent element of the ‘intensive mothering ideology' is presented in the idea that children deserve the presence of their mothers throughout the day, reflecting the traditional notion of women being responsible for the home and men being the breadwinners. As such, this ideal can evoke feelings of guilt or insufficiency for those alternating between the working and the family sphere. Of our participants, six out of 17 women worked at least partly in home office due to the pandemic and had not done so before. Vanessa states that the current situation leads to feeling overwhelmed and not enough, as she more strongly than ever thinks that she is not doing justice to her job- and mothering-related tasks (Vanessa, I: 7). The feeling of overload is similarly experienced by Julie, who expressed high stress as she is “torn between raising children and home office and still can't really do justice to either side” (Julie, I: 16). Bianca deals with exhaustion and guilt as she needs to prioritize her work over her son:

“This is strenuous so that I am often very exhausted after work (...). During working hours, I often have a guilty conscience, because I would like to take care of my son more” (Bianca, I: 17).

Because of having to perform simultaneously as mothers and workers, Vanessa, Bianca, and Julie face a role conflict in deciding which of the competing demands to prioritize. Experiencing this stalemate makes these women feel doubtful, failing, and guilty.

#### Juxtaposition of mothering and working II: Balancing identities

A different situation is presented by Helen and Sofie, who emphasize how privileged and thankful they are for having a house, garden, and flexible working options. The different expectations toward their “mothering” and their “working” identity are experienced as less conflicting and easier to reconcile. Due to her management position, Sofie can bring her children to the office whenever she needs to be there and work from home the rest of the time. This flexibility allows her to split up her work into reasonable time slots and, in parallel, spend time with her children (Sofie, I: 8). Helen and her partner also work flexibly from home and enjoy having additional time as a family. Both women neither articulate feelings of guilt or self-doubt related to their mothering, nor do they feel overly stressed. The “intensive mothering” norms that propagate an incompatibility between being a “good” mother and participating in the labor market do not seem to affect their maternal self-conception.

### References to the “ideal worker” norms

As alluded to in the previous paragraphs, we will now turn to how some interviewees implicitly referred to the “ideal worker ideology.” In five interviews, we identified three different types of references to this ideology: first, constructing the male partner as “ideal worker” (*n* = 3), second, (failing to) constructing the “ideal worker self” (*n* = 1), and third, framing the homemaker-self within the “ideal worker” ideology (*n* = 1).

#### The male partner as “ideal worker”

At the time of the interview, Mary's work had been declared as “essential,” meaning that she now can use the so-called “emergency” childcare services for the hours she is at work. The weeks before, Mary's child was cared for at home due to the general closings of childcare facilities. While Mary and her husband for 2 weeks alternated between a morning and an afternoon shift of working and taking care of the child, Mary then took 3 weeks off, explaining that it was too challenging to work and care simultaneously:

“At the beginning, organizing childcare was the biggest challenge. It quickly became obvious to us that childcare and normal work could not be reconciled, so I took leave of absence” (Mary, I: 2).

During those 3 weeks, Mary was entirely responsible for her child, and now, that her child is back in kindergarten, she is back to work. Interestingly–and matching the male “workers ideal,” it seems unquestionable that Mary (as the mother and the one in part-time occupation) is the one taking days off from her work and returning to work now that childcare is secured again.

Such maintenance of the male ideal worker is also apparent in Lea's interview. Lea fully has her husband's back so he can pursue his work during regular hours. In contrast, she shifts her working hours into the early mornings, late evenings, the nap times of her younger child, and the weekends. She states that occasionally it puts pressure on her to only have disrupted time slots to work, yet she does not see a realistic possibility to change the current arrangement:

“In particular, I find the home office sometimes burdensome, because my husband works his 100% job at his desired time (…). I work reduced (…) hours (60%) and make sure that I always find time slots for it or work on the weekend. Sometimes I find that unfair (…). I would like my husband to also work on one day of the weekend and, for example, on two evenings, so that I could also work during (…) the morning. But because of his work or the (…) video conferences with colleagues, this is not so easy to implement” (Lea, I: 10).

Due to her part-time employment compared to her husband's full-time job, Lea justifies and creates the conditions for his continuation of regular working hours. By doing so, she maintains her husband's ideal worker status at the expense of her own work-related needs. Due to her position as a teacher, Lea will soon be allowed to use the childcare facilities. Just like Mary, Lea justifies her children returning to kindergarten with the increase of time that she will have to spend in presence at school:

“In May [2020], the situation will probably change a bit, as I will then have more attendance time at school again. Our children will then (have to) visit the emergency care of the daycare center for these times since we cannot organize the care otherwise.” (Lea, I: 10).

Both women justify the return of their children to childcare facilities exclusively by the changes in their own work conditions. For their partners, nothing substantially (except for now working remotely) seems to have changed: their working hours are still during the day and predominantly uninterrupted. This shows that the worker ideology not only leads to the prementioned continuation of their husbands as “ideal workers” but also how they recur to this ideology by axiomatically taking the additional care hours into their sphere of responsibility by taking days off (Mary) and shifting work to the children's sleeping times (Lea).

Kate reports a similarly disproportionate distribution of care work at her expense. While her husband works in “home office from early in the morning until the evening, [she is] from 8:30 to 14:30 (…) in the home office, while in parallel being responsible for [her] daughter's schoolwork, entertain [her] son and [conjuring] up a lunch” (Kate, I: 13). She feels hugely stressed by the multiple and competing demands she currently experiences:

“The multi-load due to home office, homeschooling, lack of daycare, the extra demands on the household (lunch every day, more cleaning,...), the extra demands on shopping (When does it make sense in terms of time? Who looks after the children during this time?...) puts an extreme strain on me. I feel like I can't get everything organized anymore” (Kate, I: 13).

She narrates that even before the pandemic, her husband's job was busy, while she “only” worked part-time and “in this respect” not “yet found it unfair to be more burdened by childcare and household chores.” Since the pandemic evolved, she alone had to “compensate for the closed daycare and schools” and all “new and additional tasks” landed on her back, which she considers unfair, burdensome, and frustrating. In accordance with Mary's and Lea's partners' situation, Kate's partner continues to perform as the male ‘ideal worker', neither having to deal with care responsibilities during his working hours nor having to piece time slots together for work. Although Kate perceives their current daily arrangement as strenuously challenging and unjust, she does not scrutinize the ‘male' workers ideology as such. Instead, she adheres to it and therefore contributes to its maintenance. She relocates the problem of conflicting roles and responsibilities that she must comply with (and fails to fulfill according to her interpretation) back to her area of responsibility:

“At the moment, however, I'm asking myself whether I can keep this up for much longer and whether this balancing act is even worth it. Professionally and socially, you don't get any recognition for taking on the extra burden. Occupational development opportunities after becoming a mother are non-existent! After all, you are only available part-time and therefore only to a limited extent. No question - I still liked my job. But now the burden is simply too great. Why should I continue to take it on?” (Kate, I: 13).

This statement not only reveals her professional self-conception as not having the same “market value” after becoming a mother but also how she (and society) still perpetuate the gender system of domesticity, including its three assumptions (maternal sacrifice for the children, employers' legitimate expectation of “ideal worker” prioritization of work over family duties, and the equation of men as “ideal workers”).

#### Construction (or failure) of the “ideal worker self”

In contrast to the previously displayed interviewees, Julie explains she and her partner (more or less) equally share the additional care effort. As both work remotely during the days, they established fixed times where one of them can work without interruption (in theory, yet, reality shows differently), and the other tries to work while having the main responsibility for the household and being present for the three young aged children. The division of care responsibility between Julie and her partner during the pandemic did not arise naturally. Instead, Julie claims that:

“In the second or third week of the lockdown, there was a major discussion because my mountain of tasks had steadily increased, while my husband continued to follow his usual activities, but from my point of view was better able to ignore the extra workload than I was. Since then, our split has been mostly fair – it's a very difficult situation for all of us, but I feel we are currently a good team” (Julie, I: 16).

While Julie initially took up the pandemic childcare burden, she does not refer to the male “ideal worker” construct to justify this disparity. Instead, she traces it back to her husband's character traits (“ability to ignore”). Yet, the “worker ideal” is also present in her interview, but in relation to herself and her employer.

Julie feels highly stressed by the lack of flexibility exhibited by her employer. Although she and her colleagues are well-equipped for home-office, the company allowed remote work during the first weeks of childcare closings only “against crediting of days off” (Julie, I: 16). In exchange for working from home, she agreed upon fixed times of availability for calls with her team leader. These times are congruent with the slots when her husband takes the primary responsibility for their children. However, Julie claims that these agreements are “torpedoed (…) by [her] employer, by the fact that no consideration is given to [her] working hours and [she] then (…) answer[s] calls while building Lego (…)” (Julie, I: 16).

The times she agreed upon with her team leader “are not taken into account when scheduling appointments,” so they conflict again with her family responsibilities. Also, the company forced employees to take 2 weeks off in April. Julie perceived this solicitation as even more stressful because of the vacation covers she eventually had to take over as “systems had to continue to be maintained, and projects were not stopped” (Julie, I: 16). For her, the extra workload coming from colleagues being on leave was simply not manageable, which is why she took her vacation as

“single days to reduce the weekly working time and thus to be able to finish work a little earlier every day - since this hardly worked and thus I only turned days off into (unofficial) overtime, I am currently no longer willing to use this approach.” (Julie, I: 16).

Julie's detailed account of the company shows how the “ideal worker” ideology is placed upon the employees, even if they find themselves in a family-to-job compatibility crisis. The company's lack of concession is based upon the assumption that “ideal workers” prioritize their work obligations above the rest of their life. By claiming that Julie is no longer willing to assimilate herself to the company's approach, she breaks with the worker-roles expectations laid upon her. She therefore constructs her company as maintaining the “ideal worker norms,” expecting their employees to adhere to this norm regardless of their personal situation. In this light, Julie constructs herself as not being able nor willing to meet the “ideal worker norm” and (as quoted before) at the same time perceives herself as ‘not being a good enough' mother.

#### Framing the homemaker-self within the “ideal worker” norms

Hanna enjoys “being at home, freely dividing [her] time, and using [her] skills and strengths to benefit [her] family,” whereas her husband “enjoys his work and is happy in the provider role” (Hanna, I: 1). Accordingly, she claims that they are both satisfied “with this division/role sharing” which is “based on [their] beliefs about how [they] want to live [their] lives (…).” She pictures the gendered division of roles between them as the most functional, satisfying, and healthy system for their partnership and family. While her husband “takes the overall responsibility” and presides over the family quasi-like the “chairman of the board who is responsible and accountable,” she perceives her role as the co-leader and manager of the operative businesses of all family-related tasks and responsibilities (Hanna, I: 1). The families' role representation, accountabilities, and task division described by Hanna seamlessly link to terminologies and phrasing of corporate governance and management: she organizes, manages, and holds all the strands of the family together, while he represents the family to the outside world. The business metaphor continues in her self-representation as a mother managing the daily lives of her children during the pandemic:

“Due to the fact that the children have fewer playdates (…), I have to increasingly provide for activities (…). I spend a higher proportion of time in organizing new games, researching craft ideas, coordinating tasks and reward system.” (Hanna, I: 1).

Hanna feels challenged by the fact that the noise level is continuously high and says that encountering these situations requires “a more targeted use of soft skills from her” (Hanna, I: 1). She also experiences the effect of daily routine, and notices that her self-discipline has declined due to fewer external pressures. This manifests in the organization of upcoming events or appointments “like hosting guests for a birthday, doctor's appointments, play dates” but also in daily chores (e.g., motivate children to do their chores and schoolwork). Skipping daily structures and being more relaxed about the household organization she interprets as a sign of her “laziness” and a “source of danger,” and therefore redirects her focus back to herself, the one responsible for ensuring a functioning family life (Hanna, I: 1).

Hanna's and her partner's roles and task divisions conform with traditional gender norms and the gender system of domesticity. Interestingly, Hanna constructs and depicts her stay-at-home mothering and homemaker obligations in a narrative congruent with the ‘ideal worker' ideology adapted to her care-work: she is fully devoted to her job, has her areas of duty clearly in mind, leads and manages while making the best use of her skills, aiming to ensure the functioning of her family, even though this costs her strength. Hanna is not complaining about her children or her duties, even though she experiences the continuous “standby mode,” and lack of pauses during the pandemic lockdown as challenging. She reacts by bringing forward the need to more consciously use her abilities (“skill sets”) to manage strains. Throughout the interview, Hanna barely mentions conflicts between her partner's occupational work duties and her duties as a stay-at-home mother. Instead, the challenges she perceives exclusively originate from an extension of her regular job as the primary care person for her children. While Hanna's and her partner's traditionally gendered role division corresponds with the ideal workers ideologies basic assumptions (female caretaker, male ‘ideal worker'), she is not explicitly perpetuating this image in her interview, but rather holistically lives it without experiencing the burden of being torn between the two ideals of being a “good mother” and “good worker.” Nonetheless, she fills the “idealized mother norms” through an ‘ideal-workers ideology' within her self-conceptions as a stay-at-home mother and homemaker.

### Maternal self-conception and expression of maternal guilt: Continuity, improvement, deterioration

Our findings indicate that narratives of *intensive mothering* and *ideal worker* ideology are common among mothers in our sample when sharing their experiences in times of the early COVID-19 pandemic, albeit in varying nuances. Many of the participants refer to these societally dominant mothering narratives implicitly but also explicitly in some cases. Aiming to understand better how the pandemic-induced changes in daily life affect mothers' self-conception, expressions of maternal guilt and wellbeing, we identify three groups of women. Across the above themes identified, we also focused on statements that reveal how mothers construct their “actual selves” against an “ideal self,” including their notions of maternal guilt.

The first group, composed of three working mothers (Helen, Tina, Sophie), shows an *enhancement in maternal-self-conception* as these mothers seem to achieve their internalized conception of ideal mothering. They blossom and enjoy the everyday life changes that accompanied the early phase of the pandemic. The deceleration in daily routines positively affects their mothering as they spend more time with their children, feel closer, and have grown together as a family. These positive changes affect their maternal conception in a way that makes them feel better about fulfilling their mothering role. Their interviews are phrased in an enthusiastic and balanced tone, even when mentioning challenging situations. Overall, they articulate a high sense of wellbeing and satisfaction in the absence of any statements that can be traced back to feelings of maternal guilt.

The second group of women (Hannah, Karen, Eve) is characterized by a *continuation of maternal self-perception* showing high convergence between their pre-pandemic and pandemic times notions of motherhood. Remarkably, this group is composed of the homemakers and the unemployed women. Despite strenuous circumstances, they have overall adapted smoothly to the situation. Their self-conception as mothers has not been queried as they experience (more or less) a continuation of their pre-pandemic routines. This stability can possibly be traced back to a high level of self-efficacy that was particularly present in these women's deliberately chosen parenting approach and their value system. A continuation of maternal self-perception could also be seen in a mother (Dana) whose biggest challenge was the lack of work-related tasks, which coincided with the closure of the kindergarten. In her interview, she does not articulate a substantial discrepancy between her ‘ideal' and her ‘actual' mothering beliefs. Instead, she positively values her identity as a worker, where she seems to experience (more) self-efficacy.

The third, and largest group of mothers Dana, Julie, Bianca, Kate, Vanessa, Jane, who are all working, experiences a clear deterioration in maternal self-conception. This is specifically the case for those who experience growing discrepancies between their actual self and mothering ideals, stating that they feel (more) challenged, overloaded, emotionally strained, exhausted, or physically stretched. Their expressions reflect not only poor wellbeing and mental health but also insecurities, feelings of failure, or maternal guilt concerning their performance as mothers. Some show high awareness about how (destructive) societal norms influence their mothering ideals (and, as such, their perception of failure in the light of these ideals). For these women, their currently low maternal self-esteem evolved in the context of the pandemic-induced changes, especially as most now work in home offices with no childcare. It is striking to see how outer stressors (e.g., closing of care facilities, home office) elicit role conflicts and strains and accumulate in feelings of maternal guilt.

Using the theoretical lens of *ideal worker* norms, we identify three types through which the *ideal worker* construction is evident in the self-and partner conception and at least to some extent channeled through *intensive mothering* norms. Whereas, the first type maintains the *male* ideal worker by not posing any additional care burden on the partners working time (Mary, Lea), the second type is characterized by the female failure to meet the ideal worker norms due to the employers' inflexibility and lack of consideration for the family-specific peculiarities during the COVID-19 pandemic (Julie). Last, one woman's construction of her homemaker self can be interpreted as an inherent adaption of the ideal worker ideology within the domestic ‘female' sphere while at the same time protecting the ideal worker status of her husband (Hannah).

## Discussion

In our analysis, we see that motherhood myths persist and are experienced as burdensome when working-, childcare, and family-related responsibilities were all transferred into the private space due to pandemic measures. We were able to make visible that mothers' notions of guilt can be linked to dominant gendered discourses on motherhood, specifically the *intensive mothering* norms. Also, we have shown how the *ideal workers'* norm can intersect with and build upon the *intensive mothering* ideology and conceivably amplify the burden of experienced gender inequality for women in our sample. For one group, the internalization of these norms imposes a harmful self-evaluation and a deterioration of maternal self-conception. In contrast, another group apparently seems to have reduced the gap between their “actual” and their “ideal” self. Although the latter seems positive at first glance, this evolution should not disguise that it might be the effect of the NPIs themselves (e.g., a sudden deceleration of daily routines) that makes these women feel like “better” mothers now, whereas the intensive mothering beliefs may remain as influential as before due to their dominant, hegemonial and gender-unequal character. The aim here is not to judge the group of women who experience a (positive) continuation of maternal self-conception throughout their deliberately chosen (rather traditional) family models, but rather to question why (specifically) working mothers get judged by society, in their relationships, and not least by themselves.

Our findings align with studies that showed how the NPIs affected mothers disproportionately since they were placed at the “pandemic frontline” (2, 43, 44). As in our mixed-methods analyses of the Family study, we found indications of the unequally distributed additional care work within households at the expense of the women's mental health (reference mixed-method paper). At least for some women in our sample, these unbalanced workloads and conflicting roles are perceived as an important trigger for deteriorating mental health (e.g., increased stress levels, decreased happiness and satisfaction with oneself) and negative bodily reactions such as migraine attacks. Our findings on managing inter-role conflicts and their consequences on wellbeing are supported as a gendered phenomenon by further research as mothers during the pandemic experience role conflicts to a higher degree than fathers (45). These insights, in turn, align with evidence indicating that among mothers, both individual stress levels and parenting stress levels have significantly increased during the pandemic (46) compared to stress levels of non-parents and fathers (8, 47). At the other end of the spectrum of pandemic-related wellbeing consequences, we also found that a group of women (mainly those who do not have to manage work-family conflicts) benefited from a slower life pace and increased family time, which has also been reflected in some parts of the literature (5, 48).

So far, studies have aimed at quantifying the burden imposed on mothers by the pandemic, e.g., the number of hours spent at home and in paid employment (49), or the setbacks in productivity that have come from their commitment to the reorganization of family life (50). Our small study adds a critical in-depth analysis of socially effective and self-imposed motherhood norms, their interconnectedness with one's professional role's expectations, and their impact on wellbeing and mental health. Similarly, critical pieces started to emerge in personal reflections of women in the field of public health and associated disciplines, e.g., in auto-ethnographies and reflexive essays (51, 52). Most of these apply a feminist perspective criticizing the (at least partially reinforced) gender inequalities in parenting, care, and paid work during the pandemic. As evidenced in our findings, the value of such approaches in understanding and mitigating the risks to health posed by gender norms, and exacerbated by the pandemic, cannot be ignored. They highlight the failures of yet another myth, which suggests that “women can have it all” (53), and simultaneously, the failure of policies that turn a blind eye to entrenched gender norms and relations and do not provide an environment susceptible of soothing the conflict between ideal –and socially acceptable versions of the mother and the worker.

### Strengths and limitations

The qualitative study design allowed an in-depth analysis of the heterogeneous experiences of a rather homogenous group of women in terms of relationship and (high) socioeconomic status, ethnicity, age of children, place of residence, and NPIs. As the implications of the pandemic are highly context-specific, women with more diverse backgrounds and occupational statuses would possibly bring to light other stressors (e.g., fewer remote work options, financial concerns, confined housing) and diverging references to intensive mothering norms than our sample. For example, the experiences of single mothers (who represent almost 80% of single parents in Europe) (54) may differ substantially from those of our sample. As common for qualitative research, our sample size, although relatively large for such a study (*n* = 17), does not claim to give representative findings nor allows us to draw general statements. Despite this limitation and the fact that the development of our interview guide was not informed by theoretical considerations specifically targeting “intensive mothering beliefs” or “ideal worker norms” (these were recurred to in the phase of data analysis), we believe that our approach allowed to highlight crucial points of tensions in maternal self-conception that are likely to be exacerbated during the pandemic.

By using email interviews, we tried to give the participants as much autonomy as possible to answer our questions without disrupting their daily routine. Also, email interviews encourage the participant to reflect on and actively create thought processes as the interview takes place anachronously. However, they also require the participants to have certain reading and writing abilities and relatively easy access to an electronic device. Our sample only includes women who could respond to our survey and email interviews, potentially excluding those who felt even more challenged and stretched during the early pandemic phase. Most women in our sample were working from home and, as such, represented a relatively privileged group. The participants' response behavior varied as some replied to our questions in a survey-imitating manner (short answers) instead of narrating their thoughts. About 25% of the interviewees dropped out after the second wave of responses (not replying to the third round of questions). A certain drop-out was expected, which is why we mitigated this risk by asking the most fundamental questions in the first and second rounds of emailing.

We elaborated on the role of two influential and mutually reinforcing discourses, namely the “intensive mothering ideology” and the “ideal worker ideology.” How mothers justify their thoughts and actions by referencing these discourses shows how strongly social norms affect patterns of actions and self-conception and how their (male) partners and employers contribute to shaping those norms. It is relevant to investigate further the role of gendered norms on (mental) health and wellbeing and challenge unequally distributed expectations and responsibilities in the work- and family sphere.

## Data availability statement

The raw data supporting the conclusions of this article will be made available by the authors, upon request.

## Ethics statement

This study was reviewed and approved by the Ethics Committee of Bielefeld University (Ref. 2020-059). The patients/participants provided their written informed consent to participate in this study. Written informed consent was obtained from the individual(s) for the publication of any potentially identifiable images or data included in this article.

## Author contributions

SB-Z conceptualized the qualitative part of the Family study with CM and LW, and wrote the first draft of the paper. VN coded and analyzed the data with SB-Z. CM, LW, and OR provided substantial feedback on different versions of the manuscript. All authors contributed to the final draft. All authors contributed to the article and approved the submitted version.

## Funding

The present study has been conducted by the Gender Epidemiology Junior Research Group, funded by Bielefeld University. We acknowledge the financial support of the German Research Foundation (DFG) and the Open Access Publication Fund of Bielefeld University for the article processing charge.

## Conflict of interest

The authors declare that the research was conducted in the absence of any commercial or financial relationships that could be construed as a potential conflict of interest.

## Publisher's note

All claims expressed in this article are solely those of the authors and do not necessarily represent those of their affiliated organizations, or those of the publisher, the editors and the reviewers. Any product that may be evaluated in this article, or claim that may be made by its manufacturer, is not guaranteed or endorsed by the publisher.
